# Exploring the nature of governance at the level of implementation for health system strengthening: the DIALHS experience

**DOI:** 10.1093/heapol/czu073

**Published:** 2014-09-11

**Authors:** Vera Scott, Nikki Schaay, Patti Olckers, Nomsa Nqana, Uta Lehmann, Lucy Gilson

**Affiliations:** ^1^School of Public Health, University of the Western Cape, Bellville, South Africa, ^2^Metro District Health Services, Provincial Government of the Western Cape, Western Cape, South Africa, ^3^City Health, City of Cape Town, South Africa, ^4^Health Policy and Systems Division, School of Public Health and Family Medicine, University of Cape Town, Cape Town, South Africa and ^5^Health Economics and Systems Analysis Group, Department of Global Health and Development, London School of Hygiene and Tropical Medicine, London, UK

**Keywords:** Action research, case study, governance, health system actors, health system strengthening, implementation, reflective learning, relationships, values and norms

## Abstract

Health system governance has been recognized as a critical element of the health system strengthening agenda. To date, health governance research often focuses at national or global levels, adopting a macro-perspective that deals with governance structures, forms and principles. Little attention has been given to a micro-perspective which recognizes the role of health system actors in governance, or to considering the operational level of the health system. This article presents a South African case study of an intervention to address conflict in roles and responsibilities between multiple actors supporting service delivery at the local level, and explores the broader insights this experience generates about the nature of local health system governance. In an embedded case study, action learning and reflection theory were used to design and implement the intervention. Data in this article were drawn from minutes, observations and recorded reflections of the meetings and workshops that comprised the intervention. A theoretical governance framework was used both to understand the context of the intervention and to analyse the dimensions of governance relevant in the experience. The study shows how, through action learning and reflection, local managers in two organizations came to understand how the higher level misalignment of organizational structures and processes imposed governance constraints on them, and to see the impact this had on their organizational relationships. By re-framing the conflict as organizational, they were then able to create opportunities for staff to understand their context and participate in negotiating principles for communication and collaborative work. The result reduced conflict between staff in the two organizations, leading to improved implementation of programme support. Strengthening relationships among those working at local level by building collaborative norms and values is an important part of local health system governance for improved service delivery by multiple actors.

**KEY MESSAGES**

- Operational governance is embedded within and influenced by the organizational and system level governance arenas.
- Managers at the level of implementation have a sense-making role in understanding and communicating the organizational and system-level governance arenas to their staff.
- Managers at the level of implementation have a pivotal role in engaging the people, relationships and norms and values of the health system to implement actions to strengthen it.

## Introduction

In the last decade, there has been global interest in health system strengthening to improve health service delivery and health outcomes in low- and middle-income countries (LMICs) (Mills *et al.* 2004; Travis *et al.* 2004). Health system governance has been recognized as a crucial leverage point for wider systems strengthening (de Savigny and Adam 2009). The World Health Organization (2007, p. 3) defines health system governance as a national function entailing that ‘ensuring strategic frameworks exist and are combined with effective oversight, coalition-building, the provision of appropriate regulations and incentives, attention to system design and accountability’. The limited body of existing health governance research also often focuses at national or global levels, adopting a macro-perspective that focuses on governance structures and forms (Ruger 2007; Kaplan *et al.* 2013), principles of state–society relationships (Saltman and Ferroussier-Davis 2000; Brinkerhoff and Bossert 2008) or broad indicators for assessment (Siddiqi *et al.* 2009). The related work on health system decentralization, meanwhile, includes consideration of sub-national levels (Mills 1994; Bossert and Beauvais 2002; Mitchell and Bossert 2010), and community accountability mechanisms and processes (McCoy *et al.* 2012; Molyneux *et al.* 2012), but also tends to focus on governance form, structure or principles. Despite recognition of the importance of people in understanding health system functioning, much less attention has been given to a micro-perspective in this governance literature, i.e. to considering the individual within the system (Bossert 1998; de Davigny and Adam 2009; Sheikh *et al.* 2011). In particular, the governance role of health managers, working at the local or operational level and charged with responsibility for implementing health system strengthening efforts, is rarely considered in LMIC health systems’ literature.

Yet, broader thinking on public policy implementation demonstrates the importance of including people working at the operational level, the level of implementation, in thinking about governance. Street-level bureaucrats, defined by Lipsky (1980) as public servants who have direct dealings with citizens, are one such group of people. Although their work is ‘often highly scripted to achieve policy objectives’ (Lipsky 1980, p. xii) and their behaviour is shaped by the broader institutional contexts in which they work (Rice 2013), they still have discretionary power in how and when they act and may support or undermine policy intentions. Hill and Hupe (2009) argue, moreover, that inquiries into public sector governance require a focus not only on the ‘what’ of governance, i.e. national structures and government authority and sanctions, but also on the multiple levels of action, activities and processes that make up the collective capacity to act, i.e. the ‘how’ of governance. In policy implementation literature, governance is, therefore, recognized as being about ‘solving problems and creating opportunities, and creating the structures and processes for doing so’ (Kooiman 1999, p. 69).

Against this background, this article presents a South African case study of an intervention to address conflict in roles and responsibilities between multiple actors supporting programme implementation at the local level. It illuminates not only some of the actors and relationships at play in local health governance, but also, more broadly, the nature of operational governance at this level. The primary questions we address are: how can local-level actors overcome conflict over roles and responsibilities in order to strengthen delivery of the HIV/AIDS/sexually transmitted infections/tuberculosis (HAST) programme in Cape Town, South Africa, and what broader insights does this experience generate about the nature of local or operational health system governance? The intervention was implemented as part of the DIALHS project (District Innovation and Action Learning for Health System Development). This is a long-term action-research partnership project between the health departments of the City of Cape Town and the Provincial Government of the Western Cape and two South African universities, which is seeking to generate new understanding of health system governance.

In South Africa, as in many LMICs (Oliveira-Cruz *et al.* 2003; Atun *et al.* 2010), primary health services are still strongly organized around programmatic interventions which allow high burden disease conditions to be prioritized in services offered at community and health facility levels. Governance of these interventions and services is, moreover, shaped by the constitution (Government of South Africa 1996) which created three spheres of government, national, provincial and local government, and made health care a responsibility of all three spheres. The National Health Act (Government of South Africa 2005) sets out principles of co-operative governance between the three spheres and adopts a primary health care (PHC) approach in transforming the health system using a district health system model, as is common in other African settings (WHO Regional Office for Africa 2008; WHO Regional Office for the Eastern Mediterranean 2010). In a large metropolitan district such as Cape Town (with a population of 3.4 million), where this research was located, there is a fourth health system administrative layer, the sub-district, and local-level governance efforts must also take account of a historical legacy, the parallel delivery of PHC services by provincial and local government authorities in the same geographic area. The integration of health services has been a focus and point of tension between the two organizations and spheres of government since 1996 but has not yet been resolved in Cape Town and some of the other large metropolitan districts.

Although addressed in a particular setting, the experiences presented in this article have relevance in a range of other health system settings given the relationships among multiple actors and the potential for conflict embedded in every health system (Frenk 1994). More broadly, drawing on governance ideas from the policy implementation literature, the article contributes to the still limited literature on health policy implementation in LMICs (Gilson and Raphaely 2008; Sheikh *et al.* 2011). It also, and unusually, presents a positive experience, focused on how to strengthen implementation by reducing actor conflict, in a field that more commonly examines why implementation fails (Hill and Hupe 2009). In these ways, it adds to understanding of what local health system governance entails, including approaches to managing relationships between actors.

## Setting and background to the problem addressed

Health services in Cape Town are delivered through two organizational structures: City Health administered by local government and the Metro District Health Services (MDHSs) administered by the provincial government. In the geographic area of Cape Town, the MDHS has divided its management into four local sub-structures, while City Health has divided its management into eight sub-districts—two of which fall in each of the four MDHS sub-structures. The two sub-districts involved in this research each have a population over 400 000 and, although approximately double the size of the World Health Organization-defined concept of a district (Tarimo and Fowkes 1989), they have the same function of being the primary administrative units for managing and co-ordinating health services, community involvement and intersectoral actions for health. We retain the term ‘sub-district’ in this article so that the organograms and titles used make sense.

**Figure 1:**
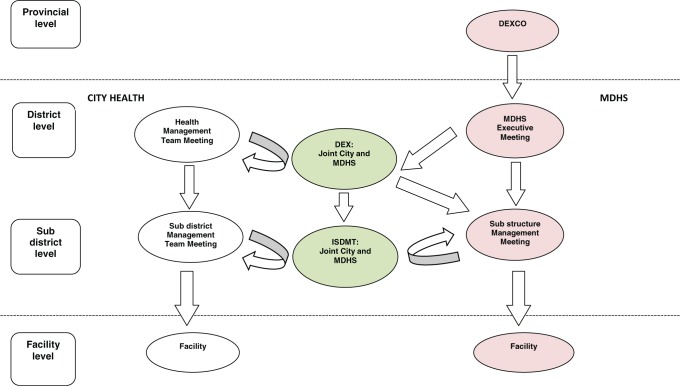
Management meetings within City Health and MDHS across the levels. Arrows indicate the flow of communication and delegation. DEXCO, Divisional Executive Committee; DEX, District Executive Committee; MDHS, Metro District Health Services; ISDMT, Integrated Sub-district Management Team.

The co-operative governance of primary level health services requires structures and processes for co-ordination and collaboration between the two organizations. City Health receives funding from MDHS for some of the services it renders and this is formalized within a service-level agreement (SLA) of primary level services (Provincial Government of the Western Cape 2010). The SLA is a contractual mechanism which structures organizational relations. Management across the levels within both health departments is achieved through a series of similarly interconnected meetings, as can be seen from Figure 1; communication, co-ordination and joint planning between the organizations is through two joint meetings.

**Figure 2:**
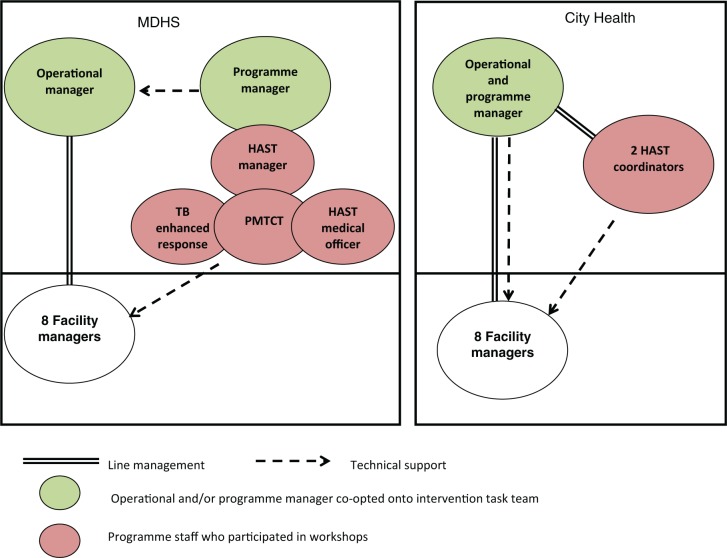
Operational and HAST programme managers and HAST staff in the two organizations. TB, Tuberculosis

HAST services are offered both within primary care facilities and in the community. The HAST programme staff do not work directly with clients and communities but are responsible for providing technical support to the staff and managers of primary care facilities and community-based organizations, and for liaising with sub-district finance, procurement and health information system staff. Figure 2 highlights the variation in numbers, titles and placements of HAST programme staff in the two organizations.

The introduction of a new management post and person in the provincial sub-structure to support the HAST programme at sub-district level within MDHS led to tensions between the organizations. Members of the research team were therefore asked for support in assisting a locally constituted task team of sub-district programme/operational managers to resolve conflict surrounding HAST-related roles and responsibilities in the sub-district.

## Methods

This research uses an embedded case design (Yin 2009). The wider DIALHS project and its context is the case (and has been described elsewhere, e.g. in Elloker *et al.* 2013) and the embedded case is this particular intervention. The DIALHS project aims to better understand, intervene in and research routine health system governance practices—learning ‘with’ rather than ‘about’ health system actors in cycles of action and reflection over a prolonged period of time. Together the team is exploring a range of issues and actions seeking, ultimately, to strengthen governance in PHC.

In keeping with the wider DIALHS project, the intervention was shaped by collaborative action learning theory, based on Rigg’s understanding of action research as ‘a collective process for inquiring into and taking action on projects and practices within their complex, multi-agent contexts’ (Rigg 2011, p. 15). As described in the Results section, participatory methods of engagement and reflective learning as the ‘purposeful critical analysis of knowledge and experience, in order to achieve deeper meaning and understanding’ (Mann *et al.* 2009, p. 123) were deliberately used in a series of meetings and workshops with stakeholders, both as method and as part of the evolving intervention to understand the causes of the presenting conflict and to identify acceptable and appropriate next steps

The intervention was followed by a phase of post-intervention analysis to allow a further cycle of reflective learning, resulting in this article. An additional task team meeting was devoted to thinking about the effect of the intervention in the sub-district. The task team identified the key moments of learning in the reflection and intervention process and translated these into themes about programme strengthening.

In order to validate these themes, the researchers, first, examined the data collected through the intervention process (which included an initial document review of agendas, minutes and observations of the task team meetings; minutes and observations of the workshops; follow-up interviews with task team members; and the researchers’ field notes) to explore how these themes of programme strengthening unfolded in the process of the intervention. Then, second, and in line with good practice for case study research (Yin 2009), the themes were validated through comparison with existing theory—Hill and Hupe’s Multiple Governance Framework (MGF) (Hill and Hupe 2009)—a framework drawn from the public administration literature. This framework was valuable for this analysis because it brought relevant insights from policy implementation literature to bear on understanding local-level health systems governance issues, including seeing these issues in relation to higher level national and organizational arrangements. In using this theory, the two researchers began by independently confirming that the themes that had emerged inductively in the reflective task team meeting were broadly supported by the MGF framework, and then, to illuminate the governance issues more clearly, as recommended by its authors (Hill and Hupe 2009), they used the framework to break down the themes into further categories corresponding to the domains within it. This analysis of the intervention was, finally, presented back to, and further developed with, two members of the task team (PO and NN). The task team members judged, in turn, that the framework not only helped make sense of their lived experience of governance and decision-making but also helped frame how, within their realm of authority, it was possible to solve problems and create opportunities for HAST staff to work together constructively

The MGF (Hill and Hupe 2009), shown in Table 1, is ‘an analytical framework that enables a structured view of the subject’, i.e. governance (Hill and Hupe 2002, p. 184). It identifies a set of nine inter-linked domains of governance action ranging across three action levels and running across three action scales: system, organization and the individual.

**Table 1:** Multiple governance framework

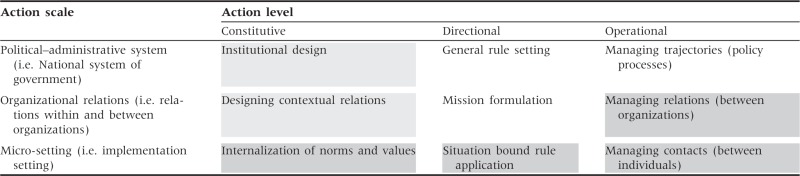

Source: Adapted from Hill and Hupe (2009).

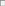
 Context of the intervention   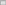 Governance dimensions in the intervention

Constitutive acts of governance are fundamental decisions about institutional design, i.e. the rules about the rules of the politico-administrative systems as a whole. Directional governance encompasses decisions about broad policy directions for government as a whole, and specific sectors such as health, including acts of prioritizing and making strategic plans. Operational governance, finally, represents decisions on how to operationalize policy and priorities, i.e. how to implement them, including managing relationships between organizational and individual agents.

Hill and Hupe (2002, p. 184) describe the political–administrative system as the action scale ‘where there is legitimate attention to and responsibility for the whole. In practical terms, this means the layer of national government and the “high institutions of state” around it’ (Hill and Hupe 2002, p. 184). This national context influences the health system through the setting of the overall rules for national and sectoral policy making and implementation, including the values and principles underpinning the system and overarching systems design issues such as the roles and responsibilities of different tiers of government. These rules influence the organizational setting and organizational relations action scale, i.e. ‘… the vertical and horizontal relations between organisations. First, the structure of the inter-governmental system is important: How many layers are there, what is the character of their legitimate authority (both general and in the case of the specific policy), and how do they relate to each other?’ *(*Hill and Hupe 2002, p. 185). The micro-setting is the action scale where individuals (including street-level bureaucrats) do the work of implementation.

Ethical clearance for the study was obtained as part of the larger DIALHS project from the authors’ institution. In line with the ethos of the broader project, the intervention was facilitated in a participatory and collegial manner. Participation in all meetings and workshops was voluntary and the researchers established ‘ground rules’ at the start of each meeting or workshop to ensure that their health service colleagues felt suitably comfortable in—what could have become—potentially conflictual engagements among the members of HAST team. Careful attention was paid to ensuring that participants’ anonymity was maintained in the documentation and write up of the study.

## Results: describing the intervention

**Figure 3:**
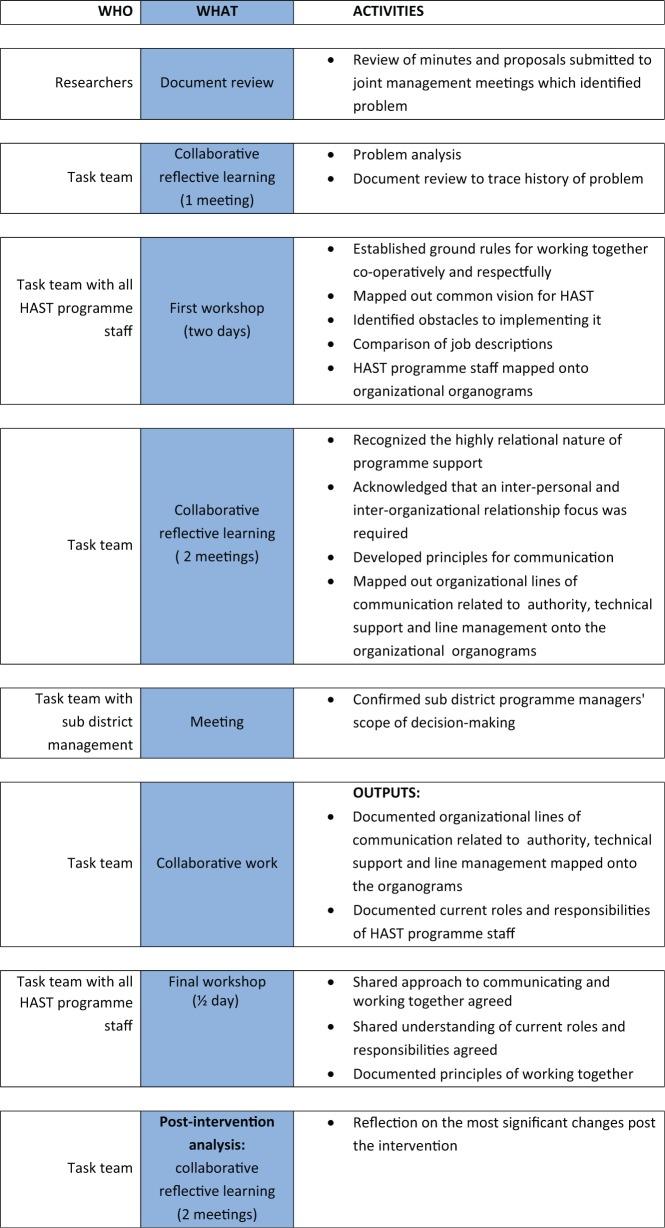
Chronology of intervention and post-intervention analysis to strengthen the HAST programme

This section describes the intervention to strengthen the HAST programme as it evolved and was implemented over an 8-month period, as outlined in Figure 3. It highlights the understandings of the underlying problems and the potential solutions that emerged and shaped the intervention.

As a first step, a task team that was established comprised five programme/operational managers responsible for management of staff offering HAST support in facilities and/or facility managers, facilitated by two DIALHS researchers (V.S., N.S.) who also documented the process and the discussions. In line with the approach of the broader DIALHS Project, the task team engaged in a process of reflective learning to develop a response (the intervention) to the presenting problem both as a small team, and in collaboration with the larger group of all HAST programme staff in the sub-district (an additional 10 staff). Within the series of task team meetings convened during the course of the intervention, the team was able to deepen its understanding of the experiences by using Moon’s (1999) stages of reflective learning—‘noticing’ and ‘making sense’ of the presenting problems to ‘making meaning [and] working with meaning’ to a stage of ‘transformative learning’ (as reflected in this article) that could serve to guide their future practice. These reflections both informed the intervention developed and provided an initial basis for the analysis of this experience presented here.

At the start of the process, a document review was conducted to identify prior expectations of roles and responsibilities and analyses of the problem from different perspectives, drawing on the minutes and written submissions made to the joint management meeting between the two organizations in which the problem was identified. The task team used this review to reflect on their own understanding and experience of the problem and what their expectations were about what ought to be resolved. Recognizing that they needed to create a similar opportunity for the broader HAST team, the task team then planned and ran a 2-day workshop for all HAST programme staff. This workshop with the full HAST staff team (managers and staff) specifically sought to understand the nature of the problem, the underlying reasons and the way forward in addressing these from the perspective of HAST staff.

The first workshop began with establishing ground rules for working together in the workshop setting and developing a joint vision for the HAST programme in the sub-district (both organizations had a strongly client-focused orientation). The main obstacles in achieving this vision were found to cluster round the following: conflict in communication; lack of respect for the organizational lines of authority; conflict over access to information in the separate health information systems to be used for monitoring and planning; inefficiencies with separate training. When it became evident that staff did not know one another’s job descriptions and relative positions within their respective organizations, these were shared in the workshop setting, giving the programme staff within each organization an opportunity to comment and ask questions. Both programme managers and staff alike had assumed that the two organizations’ structures and processes mirrored each other but, when the two organograms were mapped out, significant differences emerged which impacted on their understanding of lines of authority and the acceptable lines of communication, as well as on policy implementation pathways.

Despite this learning, it was still not possible for the HAST programme managers and staff to plan a way forward to achieve their common vision. HAST programme staff called instead for a set of standard operating procedures, approved by the district MDHS and City Health executive managers, to provide instruction on how to work together. In this they were seeking to establish rule-based relationships in working with colleagues in the partner organization and to be protected from uncertainty within these relationships by official organizational decrees.

The task team reconvened after the first workshop and, through a process of reflection, realized that HAST staff do their work with and through a wide range of stakeholders across sub-district departments and levels, and between the two organizations. In addition to mastery in technical HAST programme knowledge and skills, they required a strong set of relationship skills and a clear framework for and understanding of the relationships.

*“I think we are now doing the real work. The relationships are key to how we do our work.”* programme manager, reflective task team meeting, 13 February 2012

This insight became a guide to further work by the task team and informed the decision to strengthen support for collaborative relationships rather than defining rules for working and communicating. They recognized that a recorded description of current roles and responsibilities was useful in promoting a joint understanding of how the work was done respectively in the two organizations, but decided that communication between the HAST team and programme/operational managers, rather than a fixed agreement, was essential in maintaining the working relationship and ensuring that all responsibilities were covered. Also, they wanted roles and responsibilities to be allowed to evolve organically within the two organizations in response to changing needs. They decided the following: there could be open communication between HAST programme staff, unrestrained by lines of organizational authority; collaborative work in planning joint campaigns was desirable; and, specialist support could be offered across organizational boundaries. They agreed to informal processes of operational planning and identified the Integrated Sub-district Management Team as the appropriate local structure for formal information sharing and joint strategic planning.

A significant point of learning for the programme managers was how the differences in the two organizations’ organograms accounted for differences in the speed of policy implementation. MDHS had a strong organizational relationship with province to which it was directly accountable. Decisions taken at a monthly meeting of province and the district management were binding for MDHS, which was mandated to implement these decisions. This resulted in a faster speed and greater ease of new policy implementation in MDHS. In contrast, City Health was required to discuss any policy change requiring additional funding with City of Cape Town, and to seek funding from MDHS for any additional mandate not already funded through their SLA. This resulted in a much slower implementation, even where City Health was a willing partner supporting the policy change. These differences resulted in misunderstandings around what could be expected and what constituted a breach of the SLA contract, with the possibility of suspicion and mistrust. The programme/operational managers realized their governance role in re-framing the current conflict over roles and responsibilities as a misalignment in organizational structures rather than individual issues of personality and power. This is illustrated in the following quote:

*“Recognising the (organizational) context is very important because it means that when there is disagreement, people don’t have to be cross with one another at a personal level, but rather seeing the constraints that the organizational structures place on individuals.”* programme manager, reflective task team meeting, 13 February 2012

The task team realized that there was a disconnect between what they had been ‘tasked’ to do by their sub-district managers, and ‘what they had found necessary to do’ once they understood the nature of the conflict. Instead of negotiating a division of HAST-related roles and responsibilities between the two organizations they had, as an alternative solution, worked on understanding and building the relationships between the HAST programme staff in the two organizations. The task team called a joint meeting with their respective sub-district managers to confirm their authority to determine the content and organization of programme work and relations at this level. Understanding the scope of authority was a necessary but not sufficient step in exercising local-level governance: importantly, they then used this authority to respond to their own analysis of the presenting conflict by strengthening relationships.

The task team presented their collaborative learning back to a final workshop with all HAST programme staff. A participatory method, building on the ground rules established in the first workshop, was used to enable programme staff and managers from both organizations to develop jointly a set of principles for working together constructively. This participatory method modelled and reinforced the approach to respectful and proactive relationship building. The principles (described in Box 1) supported the internalization of trust-based values (e.g. being programme staff who are passionate, persistent and respectful in how they work) and norms to govern communication and collaborative work (such as around information sharing and proactive, constructive problem solving when obstacles arise). One programme manager later reflected that the value of these principles lay in their ability to ‘neutralize power and hierarchy’ in relations between staff who have to work together collaboratively (programme manager, reflective task team meeting, 16 May 2012). The final documentation on current roles and responsibilities, principles for working together and lines of communication was presented at the Integrated Sub-district Management Team meeting and jointly approved by the two sub-district management teams.

**Box 1. Principles developed to support working collaboratively in the HAST programme**

*Participation by all:* The active participation by ‘all’ HAST team members will be encouraged in small group discussions, committee meetings and in public forums.

*Respect of diversity:* Differences of opinion, work experience and perspectives among the HAST team members will be respected.

*Respectful communication:* HAST team members are encouraged to speak out freely, and in an honest, clear and respectful way with their HAST colleagues. In certain circumstances, and where appropriate, the established lines of authority in both the City Health and the MDHS ought to be adhered to in relation to such communication.

*Information sharing:* HAST team members are encouraged to share information with their colleagues at all levels of the health system, and to support a culture of ‘open access’ to information (e.g. welcome colleagues’ attendance at meetings, share information about a new policy). Where appropriate, this information should be shared within the context of the formal lines or pathways of communication.

*Collaboration:* HAST team members are encouraged to work collaboratively together, and in so doing provide a unified and seamless level of support to their colleagues in the health services (e.g. to the facility/clinic managers and to their staff), to the Not-for-profit organisation (NPO) stakeholders—and where appropriate, to community members. On joint initiatives (e.g. the HAST audit, a health promotion campaign, a training event) HAST team members are encouraged to share the tasks in an equitable manner and to make the best use of the skills and resources that are available both ‘within’ the respective teams and ‘between’ the two organizations.

*Problem solving:* Where problems, challenges or difficulties arise between HAST team members, or in relation to the team’s activities and programmes in the facilities or communities, HAST members are encouraged to proactively put in place a constructive problem-solving process.

*Acknowledge organizational differences:* Be mindful of the differences between the two organizations that impact on how the work is done and the speed of policy implementation. Recognize these as institutional constraints rather than personalize the problem.

*Passion and commitment in teamwork:* It is acknowledged that HAST team members are (and ideally ought to be) passionate and committed individuals who are able to use this passion to work together to improve HAST services within the respective teams and between the two organizations. In order for this to happen, a measure of self-awareness is required.

*Persistence:* Considerable persistence and patience are required given the complexity of the nature of the issues and the environment in which the HAST team seeks to work collaboratively.

In the ensuing post-intervention reflection and analysis, three core cross-cutting features were identified as important to the success of the intervention: understanding and acting to make understood the differences between the two organizations; understanding and acting to support the highly relational nature of the work of HAST staff; and developing, modelling and operationalizing a set of relational norms and values hinged on respect and valuing the ability to collaborate to deliver a client-focused service.

## Discussion: what insights does this experience offer about the nature of local governance?

In this article, we set out not only to describe the intervention as implemented but also to explore what it illuminates about the nature of local health system governance. By way of summary, Table 1 highlights, through shading, the dimensions of the MGF that have resonance in this particular experience. The darker grey cells represent dimensions of the intervention as implemented and the lighter grey cells represent contextual influences over local-level action. Table 2 then summarizes the key challenges experienced in the HAST programme and the way they were addressed through the intervention. These experiences offer two central lessons about the nature of operational governance in health systems of wider relevance.

**Table 2:** Challenges and subsequent governance action in the HAST experience

Acts of governance (Hill and Hupe 2002/2009)	Challenges experienced in the HAST programme	Governance action in the HAST programme intervention
Organizational relations		
Managing relations	Contextual constraints: Two organizations falsely assumed to work similarly Key structures (such as organograms) and policy processes were not aligned	Creating an opportunity to explore organizational differences Reframing conflict as organizational rather than personal Developing a common understanding of organograms and different policy implementation pathways Identifying and using the Integrated Sub District Management Team as the appropriate local structure for formal information sharing and joint strategic planning
Implementation setting		
Managing contacts between people	HAST staff work with a range of actors and are required to co-ordinate work across two organizations HAST staff not aware of each other’s job descriptions which led to conflict about role expectations Structures and processes for communication and developing collaborative activities were not defined	Creating awareness around the importance of people and relationships in HAST programme work Sharing job descriptions within and between organizations Developing an agreed approach to working together outside of formal processes for operational planning Granting permission for HAST staff to work across organizations
Internalization of norms and values	Antagonism between staff in two organizations	Affirming and building on the HAST programme’s common client-centred focus Agreeing on a common HAST vision Establishing principles of working together as a HAST team

First, in this case study, we see how operational governance is embedded within and influenced by the organizational and system-level governance arenas (or action scales, Table 1). Indeed, reviewing the experience through the MGF lens helped the local managers recognize the governance constraints on their actions derived from the design of the broader system and organizational setting in which they work. Despite the constitutionally established principle of co-operative governance across national, provincial and local government in South Africa, in practice the two local organizations (MDHS and City Health) have health structures and processes, such as organograms and meetings, that are not fully aligned. Recognizing these contextual constraints helped the local managers to understand the nature of the problem they were seeking to address, and then make sense of it with their staff.

Although the presenting conflict appeared to be one of personal power struggles, the managers and their staff came to recognize it as an organizational issue, reflecting the impact of organizational differences on acceptable lines of communication and authority and on the speed of policy implementation. Aligning the structures and processes of the two organizations was beyond the authority of the programme/operational managers but, in recognizing that ‘the calamity lies in “misunderstanding” the levels and roles’ (minutes of reflective task team meeting, 13 February 2012), the managers were then able to assist their staff in re-framing the conflict in terms of a misalignment between two sets of organizational structures and processes. This new framing made it possible for staff to be more open and trusting of their colleagues in the other organization and to enter a meaningful dialogue about how to work together.

Health decentralization literature (Collins 1996) similarly considers how, within bureaucratic environments, higher tiers of government influence the decision-making space (governance) of lower tiers through delegating power and responsibility. However, the exploration of governance in this article goes beyond a concern for the ‘scope of decision-making’ at different levels to examine broader dimensions of how the organizational context shapes the local level. In doing so, it moves beyond the common focus on authority and rules in health systems literature to consider how to work through other ‘modes of operational governance’ (Hill and Hupe 2009), and to engage the people and relationships of the health system in implementing actions to strengthen it. In this way, the article adds to the small body of LMIC literature that demonstrates the importance of engaging people and relationships in managerial action to strengthen health systems (McIntyre and Klugman 2003; Walker and Gilson 2004; Crook and Ayee 2006; Kamuzora and Gilson 2007; Scott *et al.* 2012; Lehmann and Gilson 2013). It also shows how local actors own understanding of their governance context can influence their behaviour.

Second, more specifically, the intervention shows how local health managers can, in the MGF language, pro-actively ‘manage contacts’ by valuing and building relationships strongly guided by collaborative values and norms, while still taking account of locally relevant rules. Once the HAST programme managers became aware of the need to support relationships between people within and between the organizations, they took deliberate steps to create an awareness of the importance of relational skills in HAST support work and to value these skills in the workshop discourse and in their ensuing management practice. This shifted the mindset of HAST staff who had previously understood their work to be predominantly technical in nature, to acknowledge that their work required good relationship with all stakeholders and that they needed relationship skills to accomplish their work successfully. The HAST programme managers then also provided spaces for the development of these relationships by creating the opportunity in workshops for staff to participate in developing shared understandings of job descriptions and organograms, and negotiating a set of principles for communication and collaborative work outside the workshops. In applying the MGF in the post-intervention analysis, the managers saw the governance dimension of this work on relationships: how it created opportunities for staff to work together effectively to support programme delivery. This resulted in them developing a broader understanding of their local governance role, one which included enabling effective relationships.

A key challenge for local programme implementation had been that structures and processes for communication and developing collaborative activities between the two organizations were not defined. The initial intention of the intervention was, therefore, to facilitate an agreement with the HAST staff on the division of roles and responsibilities between the two organizations. In practice, however, the work on understanding respective job descriptions and organograms, together with developing a set of principles for working together, eliminated the need for formal agreement. Rather than spending time defining roles and responsibilities locally, which would inevitably have to change as needs change, the HAST team recognized the importance of developing principles that could guide them in how to respond collectively to new situations over time. To support the internalization of these principles, the programme/operational managers role modelled the values by using respectful and participatory methods of problem solving in the workshops and by demonstrating them through their own work in the task team.

Although the theoretical health governance literature recognizes a range of influences over behaviour (Brinkerhoff and Bossert 2013), a common focus in much of the decentralization literature is, essentially, on the different configurations of authority embedded in different forms of decentralization (Mills 1994). Drawing on principal-agency theory, the decision-space framework (Bossert 1998; Bossert and Beauvais 2002) adds to this literature by considering the economic and political incentives influencing local actor decision-making within decentralized systems. Hill and Hupe (2009), however, go further in recognizing three stylized modes of governance, or management approaches, that can operate in parallel within any system. In the authoritarian mode of governance, compliance with instructions and rules is the management mechanism; in the transactional mode, management via incentives and contracts is emphasized; and, in the persuasive mode, characterized by co-production, building trust is important.

In the embedded case study reported here, the principle of co-operative governance embedded in the overarching design of the South African political and health system was brought alive locally through the shared development of norms and values to enable trusting relationships and guide collective action. Understanding the rules shaping individuals’ behaviour (roles and responsibilities) was important but not enough to support the collective action necessary to strengthen HAST services. Importantly also, the shared development of these principles by HAST staff and their role modelling by local managers were seen as assisting their internalization by local actors. These actions show, therefore, how to operationalize the normative principles highlighted in macro-governance frameworks (Saltman and Ferroussier-Davis 2000; Brinkerhoff and Bossert 2013).

Eight months after the last intervention workshop, a further cycle of reflection with the task team identified evidence of the continued effect of the intervention as a whole. These included several occasions in which HAST programme staff in MDHS and City Health had worked well together in supporting service delivery implementation in the sub-district: in planning a joint campaign, in supporting training and in giving direct technical support to facilities across organizations. In addition, the task team reported that the HAST-specific intervention appeared to have had positive repercussions for other health programmes within the sub-district. For example, the greater understanding of organizational differences and needs had enabled nurses who had been trained in child health to be placed in the partner organization for a period of experience and mentorship. It was noted that ‘working together [in HAST] had spilled over into other areas’ and allowed for greater collaboration between the two organizations (reflective task team meeting, 16 April 2013).

This approach (valuing and building relationships based on understanding organizational differences and developing values and principles for working together), therefore, offers useful insights for local managers in other settings where conflict might arise in relationships among the multiple actors supporting or responsible for delivering services (Frenk 1994; McIntyre and Klugman 2003; Blaauw *et al.* 2004; Shigayeva *et al.* 2010). As in South Africa, these include relationships between different government authorities (at national or sub-national levels), across levels of the health system (such as national, provincial or state, and district) or between dedicated health programmes operating in parallel to each other and to those primarily responsible for general service delivery. However, it is important to note that the approach implemented in this experience requires managers who are willing and able to engage with each other, be reflective and learn together across actor groups.

Overall, the intervention discussed here embodies the understanding of governance carried in Kooiman’s definition (presented in the introduction). Through the ‘processes’ of the intervention (meetings and workshops), the local [programme/operational] managers engaged in ‘problem solving’ when they sought to understand the obstacles and underlying reasons for these obstacles. They also ‘created opportunities’ for programme strengthening in how they brought programme staff together to develop joint understanding, how they reframed the conflict and then oversaw the joint development of principles for working together. The ‘creation of structures’ was, however, not a feature of this intervention, because the structural change required was beyond the local managers’ level of authority. Nonetheless, these managers found that developing shared understanding of the organizational context and principles of working together was sufficient to foster trust and build relationships to strengthen programme implementation. Rather than simply defining roles and responsibilities at one time, this approach supported communication and collaborative relations with the intention of allowing roles and responsibilities to evolve over time in response to changing need.

## Conclusion

In this case study, the use of a participatory action research approach enabled the task team to address the governance constraints under which they had to solve their problem in an innovative and flexible way. Reflective practice proved to be a valuable learning approach for the local managers, allowing them to notice and understand problems and find new opportunities in a responsive manner. In designing the intervention, local managers chose to use participatory methods to involve staff in the learning and decision-making.

The experience reported here contributes an empirical case study to the existing, often quite theoretical or normative, literature on health governance. The use of the MGF as an analytical lens allowed the nature of operational governance in a local-level setting to be explored. It supported a micro-level governance focus on actors, relationships and ways of managing them that recognized the particular institutional context in which they were embedded, in contrast to the more macro-level focus of much other health governance research.

Finally, the case study suggests that people-centred governance must start by appreciating that people work together within relationships (both individual and organizational) and must pay attention to these relationships and the values and norms that underlie them. This then allows the development of health system strengthening activities that are grounded in local people and relations.
